# Effect of EPS geofoam inclusion parameters on the structural performance of rigid box culverts under induced trench installation

**DOI:** 10.1038/s41598-026-59860-6

**Published:** 2026-06-30

**Authors:** Mamdouh Eldamarawy, Mohamed Medhat, Mohammed Rabie, Hussein Mostafa

**Affiliations:** 1 Faculty of Engineering at Mataria branch, Capital University (formerly Helwan), Cairo, Egypt; 2 Construction Research Institute, National Water Research Centre, El Qalyubia, Egypt

**Keywords:** Buried rigid culverts, EPS geofoam, Induced trench installation, Soil arching, Stress redistribution, Engineering, Environmental sciences, Materials science

## Abstract

Buried rigid box culverts are widely used in transportation and water structures and are often constructed beneath high embankments. Due to the stiffness contrast between the rigid structure and surrounding backfill soil, the soil column directly above the culvert experiences smaller settlement than adjacent soil columns, which may result in stress concentration and increased vertical pressure on the culvert. The induced trench installation (ITI) method was introduced to mitigate this problem by adopting a compressible inclusion above the structure to initiate positive soil arching and redistribute the loads away from the culvert. This study investigates the influence of expanded polystyrene (EPS) geofoam inclusion parameters on the structural behavior of buried rigid box culverts through a series of experimental tests. Eleven reduced-scale laboratory model tests were conducted, including one reference test without EPS and ten tests incorporating EPS inclusions with varying densities, thicknesses, widths, and installation locations. Static surface loading ranging from 20 to 140 kPa was applied using a rigid footing system, while vertical pressures within the backfill were monitored using miniature pressure sensors installed above and beside the culvert. The results show that EPS inclusion significantly alters the load transfer mechanism within the backfill and promotes the development of positive soil arching. Among the investigated parameters, EPS thickness had the most pronounced influence on stress reduction, increasing the pressure reduction efficiency from approximately 50% to about 70% as the thickness increased from 2.5 cm to 10 cm. A lower EPS density also slightly enhanced stress reduction due to its higher compressibility. The findings demonstrate that properly configured EPS geofoam inclusions can effectively reduce vertical stresses acting on buried rigid culverts and improve their structural performance under embankment loading conditions.

## Introduction

Buried rigid box culverts are essential components of transportation networks and water structure systems. Often these structures are installed beneath roadway embankments to convey drainage flows, irrigation water, roads, railways, and underground utilities. In many situations, culverts are constructed under high embankment fills, where the interaction between the rigid structure and the surrounding soil governs the load transfer mechanism within the backfill. The rigid culverts have significantly higher stiffness than the surrounding soil, the soil column directly above the structure typically experiences smaller settlements than the adjacent soil columns. This difference in deformation results in stress concentration above the culvert, which may lead to structural cracking, excessive deformation, or long-term serviceability problems if not properly accounted for during design^1^. The mechanism controlling load transfer in buried conduits was first described by the classical theory proposed by Marston and Anderson^2^. According to this theory, the distribution of vertical earth pressure acting on buried structures depends primarily on the relative movement between the soil column above the structure and the surrounding soil columns. When the structure settles less than the surrounding soil mass, negative soil arching develops, resulting in additional loads being transferred to the structure. Conversely, when the soil column above the structure experiences greater settlement than the adjacent soil columns, positive soil arching is mobilized. Since a part of the overburden load is redistributed away from the structure toward the surrounding soil. This soil arching mechanism plays a fundamental role in determining the earth pressure distribution around the buried structures^3,4^. Several design approaches have been proposed to mitigate the risk of the increase in the overburden pressure above these buried culverts. One of the most widely adopted approaches is the induced trench installation (ITI) method. In this method, a compressible inclusion is placed above the culvert to reduce the stiffness of the soil column directly above the structure relative to the surrounding soil. This difference in stiffness enables a differential settlement within the backfill soil in opposite direction and facilitates the mobilization of positive soil arching, which results in a reduction of the vertical stresses acting on the culvert^5,6^. Experimental and numerical investigations have demonstrated that the stress distribution around culverts installed using the induced trench method differs significantly from that observed in conventional positive projecting installations. Centrifuge tests and numerical analyses conducted on buried box culverts have indicated the mechanisms governing induced trench installations. These studies have indicated that the presence of a compressible zone above the culvert can significantly affect the load transfer mechanism within the backfill soil and reduce the magnitude of the overburden pressure acting on the structure^7^. Also, field measurements obtained from instrumented culverts constructed beneath high embankments have also confirmed that induced trench installations can effectively reduce the loads acting on buried culverts. Furthermore, these investigations have indicated that stress redistribution within the backfill may influence the pressures acting along the culvert sidewalls and base owing to the development of drag forces along the structure boundaries^1^. Consequently, a comprehensive understanding of the soil–structure interaction mechanisms governing induced trench installations is necessary to ensure the safe and economic design of buried culverts^1^. In recent decades, compressible inclusions composed of expanded polystyrene (EPS) geofoam have been increasingly used in geotechnical engineering applications to modify stress transfer mechanisms and control differential settlements. EPS geofoam is defined as a lightweight cellular material characterized by extremely low density, high compressibility, and favorable mechanical properties, which make it particularly suitable for use as a compressible inclusion in soil–structure systems^8^. EPS geofoam strength is strongly dependent on its density and usually exhibits a nonlinear stress-strain behavior. Accordingly, EPS geofoams have been widely adopted in various geotechnical applications including embankment construction, bridge abutments, retaining structures, and underground infrastructure systems^9^. Several studies have indicated the effectiveness of EPS geofoam inclusions in reducing the vertical stresses acting on buried structures. Experimental investigations on rigid box structures adopting EPS inclusions indicated that the configuration and mechanical properties of the geofoam layer significantly influence the redistribution of the pressures above the structure^10^. Also, research focusing on shallow tunnels indicated reductions in vertical stresses ranging approximately between 35% and 50% depending on the thickness of the compressible inclusion and the degree of soil arching mobilized within the surrounding soil^8^. In addition, analytical solutions developed for culvert systems under high embankments highlighted the importance of the stiffness contrast between the backfill soil and the compressible inclusion layers in controlling the magnitude of vertical earth pressure acting on buried structures^11^. Several numerical studies have introduced the behavior of buried structures with compressible inclusions. Finite element analyses have demonstrated that EPS geofoam layers can significantly change the stress distribution within the backfill soil and improve the structural performance by reducing the loads transferred to the structure^12^. Furthermore, investigations focusing on soil arching mechanisms have shown that the efficiency of compressible inclusions depends strongly on the stiffness contrast between the soil and the inclusion material, as well as on the geometric configuration of the compressible zone within the embankment. Despite the conducted research considering the presence of compressible inclusions with buried structures, several aspects remain insufficiently investigated. Since most previous studies have primarily focused on evaluating the influence of individual EPS parameters, such as thickness or width, or on comparing induced trench installations with conventional culvert installations. However, the combined influence of EPS geofoam parameters, including density, thickness, width, and location relative to the culvert, has not been comprehensively examined through controlled experimental investigations^13^. Understanding the influence of these parameters is essential since they directly affect the stiffness contrast between the compressible inclusion and the surrounding soil mass and consequently control the development of soil arching within the backfill. Variations in the EPS density could directly affect the compressibility and stiffness of the inclusion material. While changes in thickness, width, and installation location determine the magnitude of the differential settlement between the soil column above the culvert and the adjacent soil columns. These parameters therefore play a key role in controlling the redistribution of stresses within the embankment and the efficiency of load reduction acting on buried structures^14^. Consequently, the objective of the present study is to experimentally investigate the influence of EPS geofoam inclusion parameters on the structural performance of buried rigid box culverts installed using the induced trench method. A series of reduced-scale laboratory model tests were conducted to evaluate the effects of EPS density, thickness, width, and installation location on the vertical stress distribution within the backfill soil and above the culvert. The experimental results provide further insight into the mechanisms governing soil arching and stress redistribution in induced trench installations and contribute to improving the design of buried rigid culverts subjected to high embankment loading conditions.

## Experimental setup

### Model configurations

**Fig. 1:**
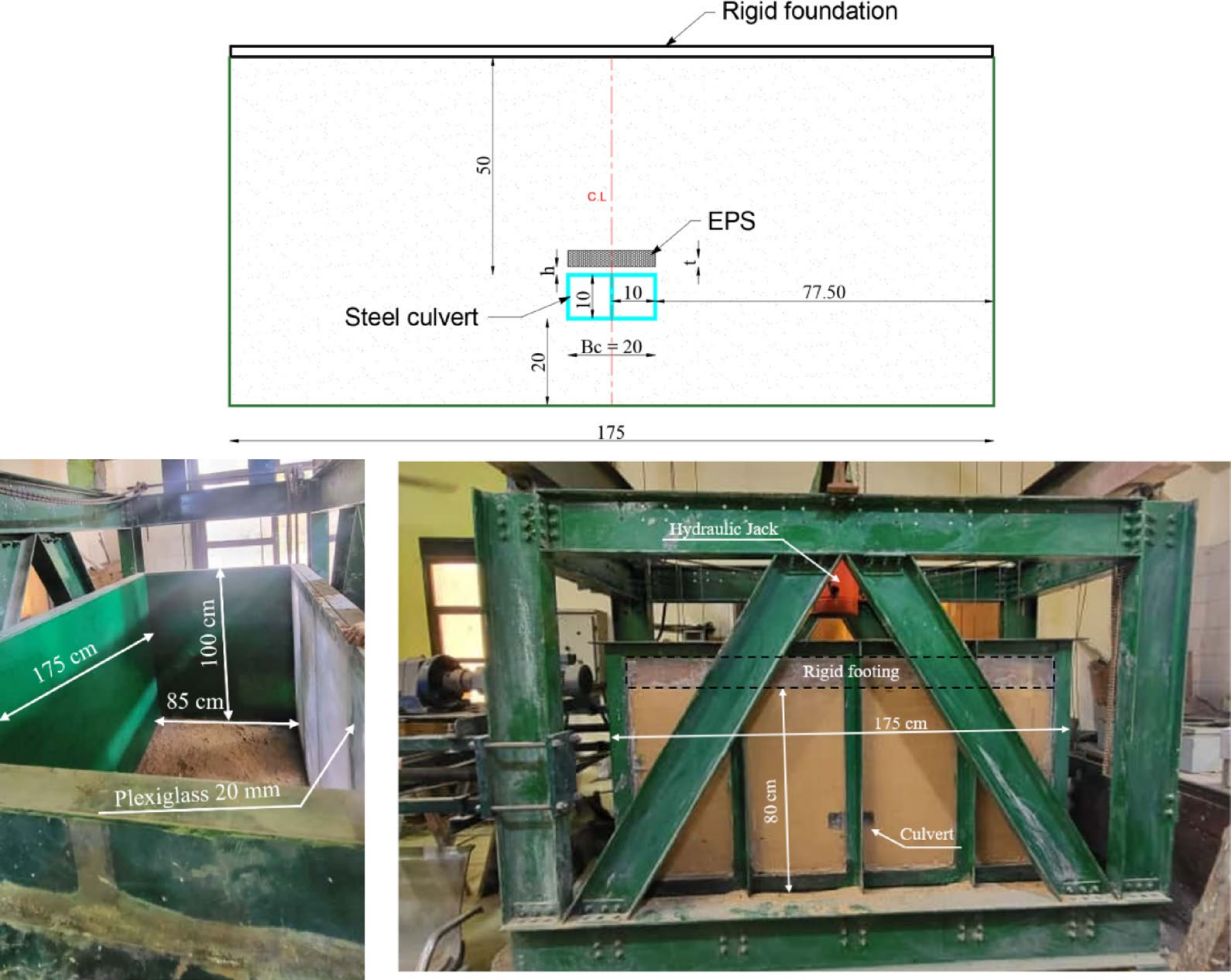
Dimensions of the test chamber.

A series of laboratory model tests was conducted to investigate the behavior of buried rigid box culverts subjected to static surface loading. The tests experimentally evaluated the influence of EPS geofoam inclusions on stress redistribution within the backfill soil. The internal dimensions of the experimental chamber were 175 cm in length, 85 cm in width, and 100 cm in height, as shown in Fig. 1. The chamber was made of three sides of braced steel and a 20 mm plexiglass on the front side to allow visual observation of soil deformations during the test. In order to minimize the friction between the soil and the internal tank steel sides, a green plastic paint was used as a lubricant. While, the plexiglass side was lifted with no treatment to enable visual observation of the soil movement.

**Fig. 2:**
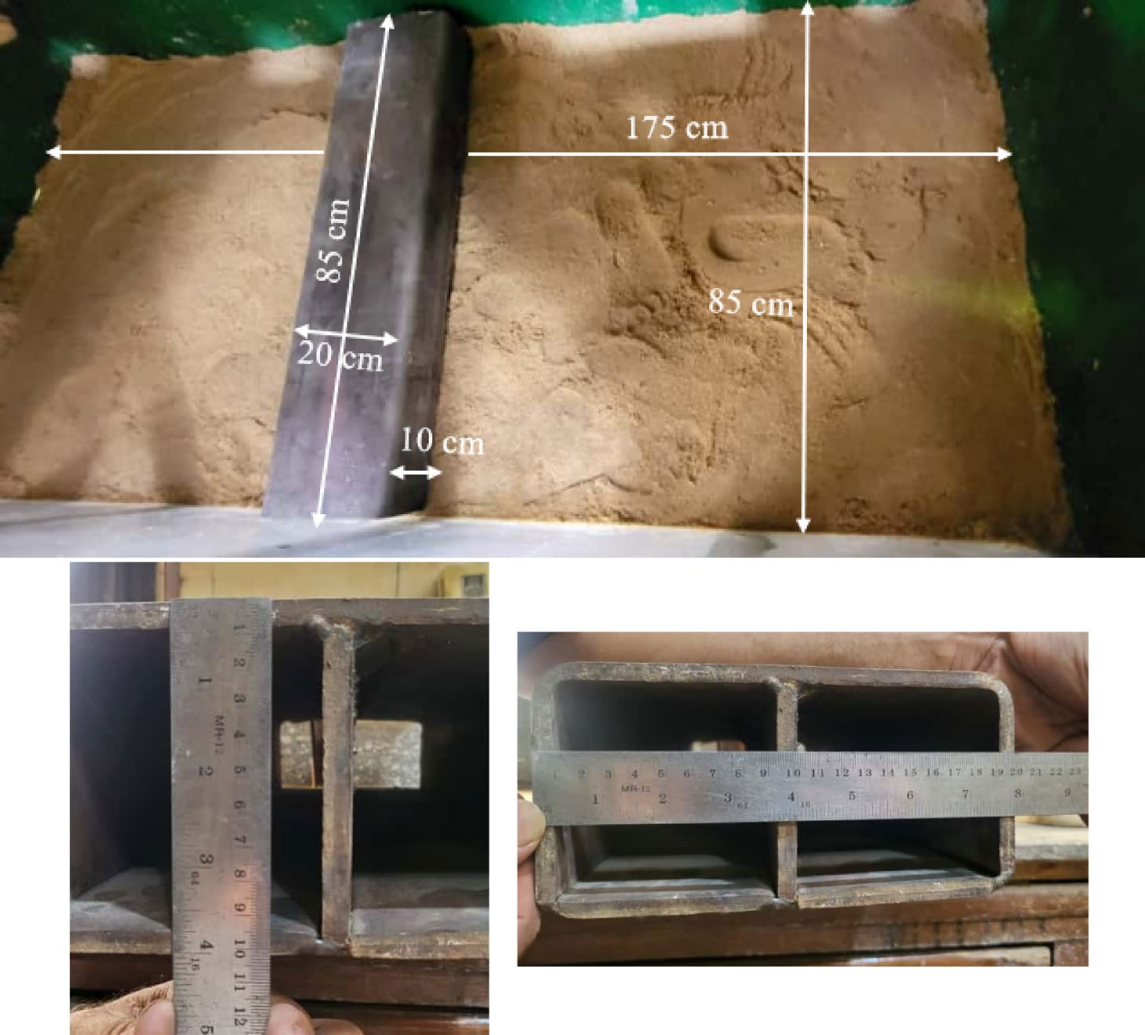
Tested rigid culvert dimensions.

A double-cell box culvert was constructed using steel plates and placed inside the chamber to simulate the rigid buried culvert structure. The culvert dimensions were a width (Bc) of 20 cm and a height (Hc) of 10 cm, and it was placed at the center of the test chamber, as shown in Fig. 2. The culvert dimensions were chosen to represent a double-cell culvert with an opening width of 2.00 m, considering a scale factor of 20. The prototype culvert width, Bc = 20 cm, was selected to be one-ninth of the test box width (i.e., the culvert was placed far from the sidewalls) to minimize the boundary effects. Accordingly, the distance from each side of the culvert to the box sidewall was 77.5 cm, which is more than twice the width of the buried structure, as recommended by^15^.

### Materials

**Fig. 3:**
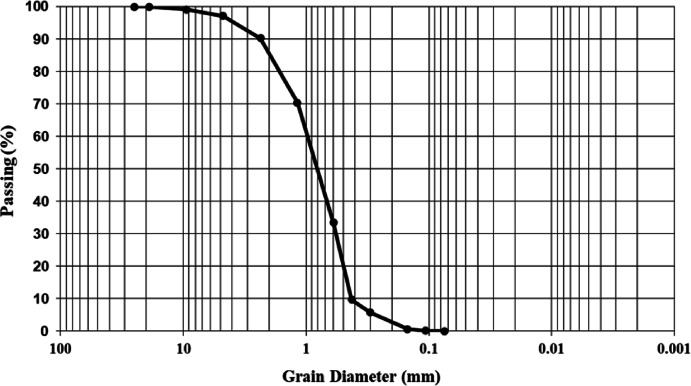
Grain size distribution curve of sand.

The backfill material was coarse to fine sand, which is typically adopted as a backfill material. The grain size distribution curve shown in Fig. 3 indicates that the characteristic particle diameters are D_10_ = 0.45 mm, D_30_ = 0.60 mm, D_50_ = 0.80 mm and D_60_ = 1.00 mm. Accordingly, the calculated uniformity coefficient C_u_ was 2.2 and the coefficient of curvature C_c_ was 0.8, indicating that the soil was poorly graded sand (SP) according to the ASTM D2487^16^ classification. The maximum and minimum dry unit weights of the sand were determined using a vibratory table in accordance with ASTM D4254^17^ providing γ_max_ = 1.83 g/cm³ and γ_min_ = 1.53 g/cm³, respectively. The maximum dry density of the sand was γ_drymax_ = 1.95 g/cm^3^, as determined according to ASTM 1557^18^. The sand backfill layers below, adjacent to, and above the culvert were placed on layers not more than 10 cm in thickness and compacted to 95% of the maximum dry density. Expanded Polystyrene (EPS) geofoam blocks were used as compressible inclusions to modify the stiffness above the culvert and to mobilize positive soil arching. The influence of geofoam on load redistribution mechanisms above the culvert was investigated by adopting three nominal EPS densities, namely, 25, 30, and 35 kg/m³. The properties of the EPS blocks are presented in Table 1. EPS blocks with different geometric configurations in terms of thickness, width, and placement location relative to the culvert top were selected to examine the effects of these parameters on the structural response.

**Table 1:** Physical properties of the EPS.

Geofoam density	EPS25	EPS30	EPS35
Compressive strength at 1% (kPa)	37	41	47
Compressive strength at 5% (kPa)	156	168	190
Compressive strength at 10% (kPa)	173	184	210
Initial modulus of elasticity Ei (kPa)	4120	4252	4540

### Test matrix and instrumentation

Eleven reduced-scale models were executed. One reference test was conducted with only a rigid steel culvert without EPS to simulate positive projection installation above the culvert. Ten tests were conducted, including EPS inclusions with varying width, thickness, installation location above the culvert, and density to simulate the induced trench installation (ITI) method, as outlined in Table 2.

**Table 2:** Experimental test program.

Test ID	Description	EPS geofoam			
		γ (kg/m^3^)	Width (cm)	Thickness (cm)	Distance above the culvert (cm)
T-00	Reference test	No geofoam			
T-01	Induced trench installation	30	Bc (20)	0.125Bc (2.5)	−
T-02		25	Bc (20)	0.125Bc (2.5)	−
T-03		35	Bc (20)	0.125Bc (2.5)	−
T-04		30	Bc (20)	0.25Bc (5)	−
T-05		30	Bc (20)	0.375Bc (7.5)	−
T-06		30	Bc (20)	0.50Bc (10)	−
T-07		30	Bc (20)	0.125Bc (2.5)	0.5Bc (10)
T-08		30	Bc (20)	0.125Bc (2.5)	Bc (20)
T-09		30	1.5Bc (30)	0.125Bc (2.5)	−
T-10		30	2Bc (40)	0.125Bc (2.5)	−

Where: Bc is the culvert outside width

**Fig. 4:**
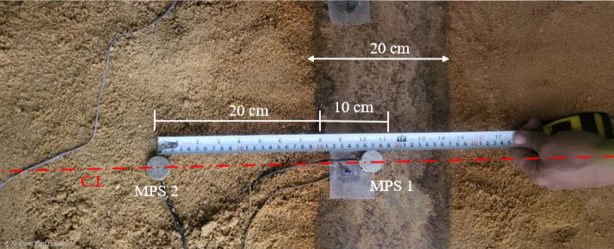
Location of miniature pressure sensor (MPS).

The overburden of vertical pressure within the backfill soil was monitored by miniature pressure cells installed at two locations, as shown in Fig. 4. The first miniature pressure cell (MPS1) was positioned directly above the culvert top slab centerline, where the maximum vertical pressure was expected to occur. The second sensor (MPS2) was installed at a horizontal distance approximately equal to the culvert width from the culvert edge to measure the pressure within the surrounding soil mass. This configuration allowed for the evaluation of the differential stress distribution and mobilization of soil arching between the central soil prism and adjacent soil columns.

### Loading system and test procedure

**Fig. 5:**
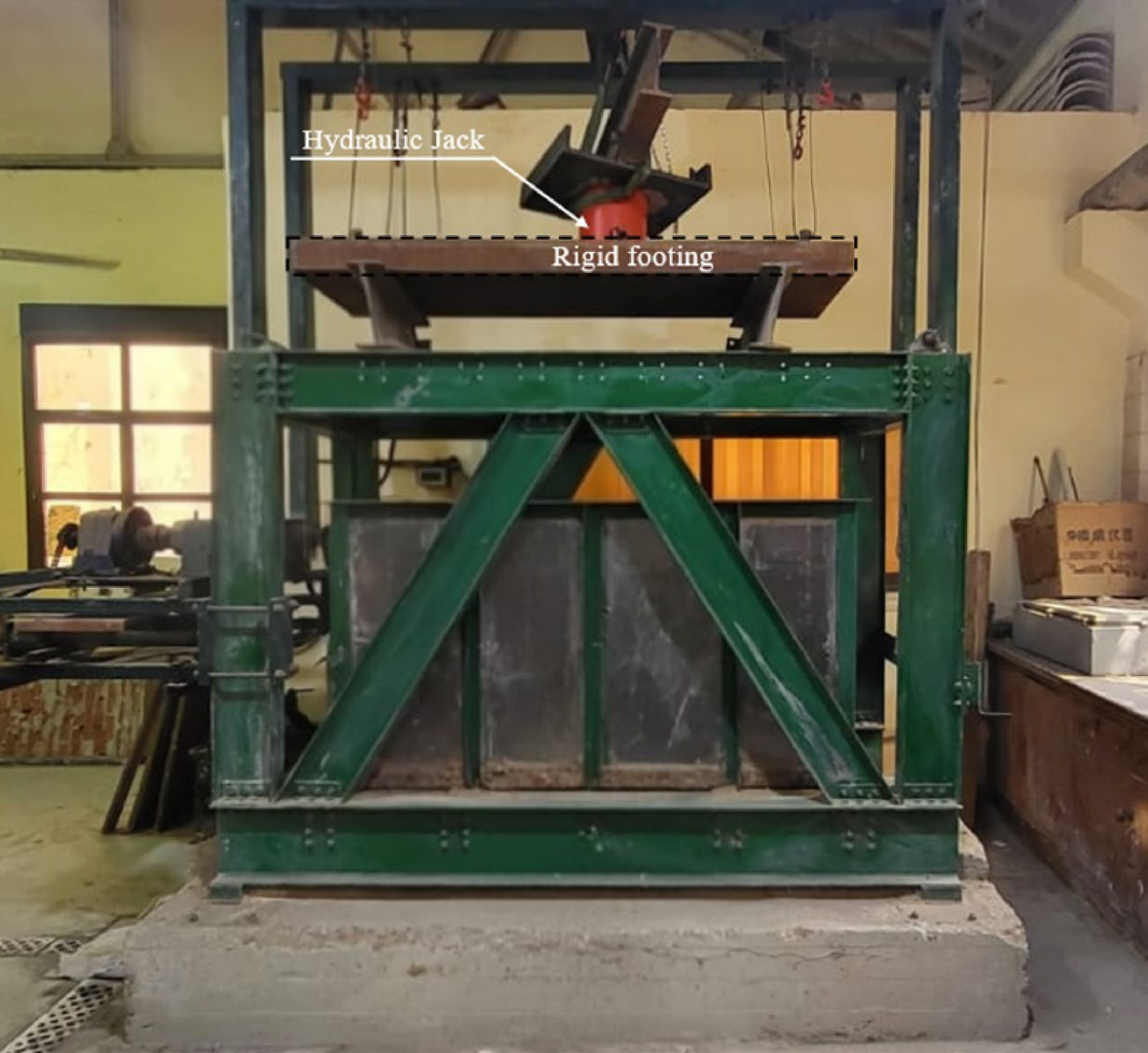
Rigid footing and hydraulic jack.

Static surface loading was applied using a rigid steel footing. The rigid footing was placed directly above the top of the backfill. Also, it had the same dimensions as the test chamber and was used to apply constant pressure on the top of the backfill. The applied load was controlled incrementally using a hydraulic loading system with a 50-ton capacity attached to a rigid reaction steel beam. The hydraulic jack and steel beam were fixed at the center of the rigid footing, as shown in Fig. 5. The applied footing pressure gradually increased from 20 to 140 kPa in increments of 20 kPa. The maximum applied foundation pressure was representing an embankment with an approximate height of up to 7.55 m. The pressure directly above the culvert was measured at the end of each loading phase after ensuring the stabilization of the soil response. The stabilization of the soil response was ensured by maintaining each load increment for approximately 15 min and until sensor readings varied by less than 1%. These incremental measures indicate the progressive development of soil arching processes and the redistribution of the vertical stress above the culvert. The same loading procedure was applied to all the tests to ensure consistent boundary and loading conditions. The comparison of the results obtained from the reference and EPS inclusion tests enabled the assessment of the influence of EPS geofoam parameters on the vertical pressure acting on the top of the buried culvert.

## Results

### Reference test without EPS

**Fig. 6:**
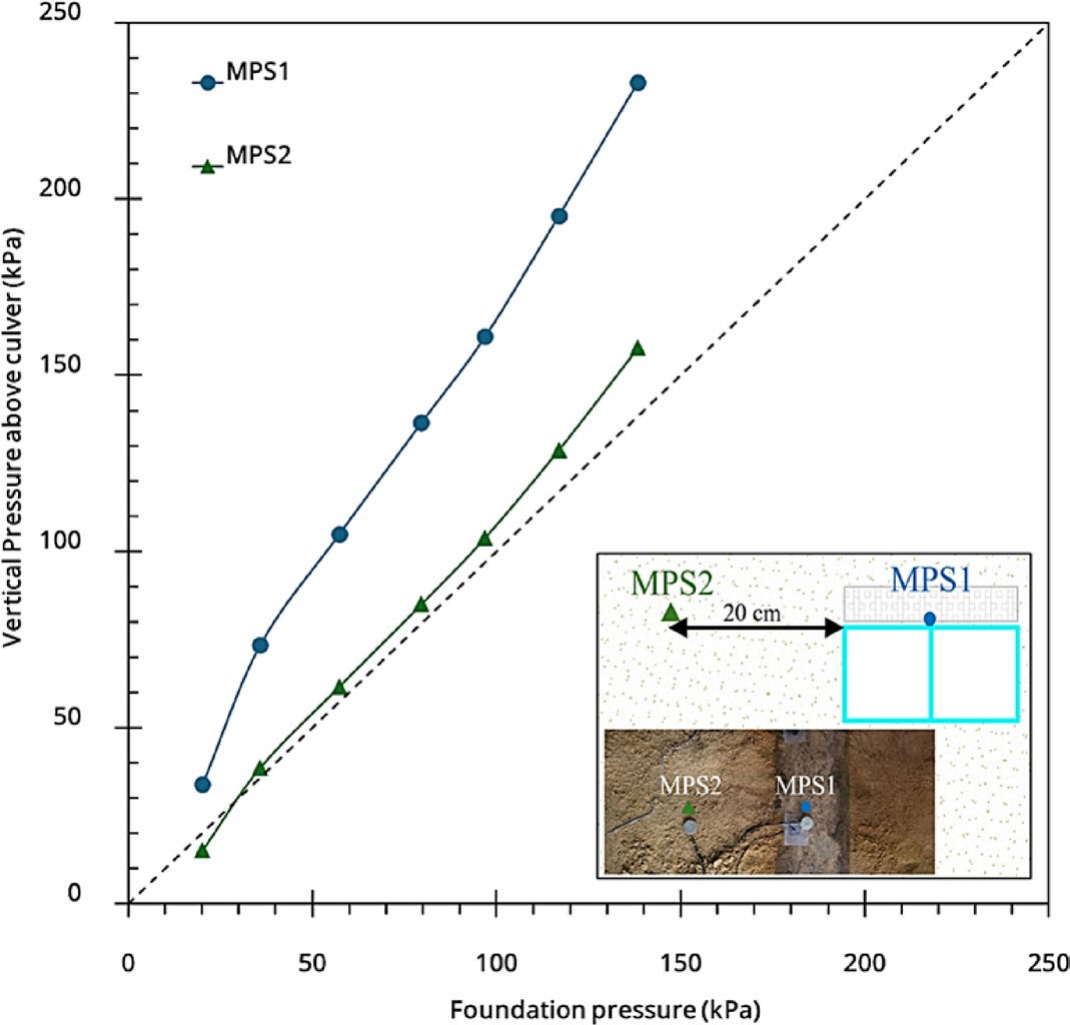
Pressure distribution above the culvert for reference test T-00.

The reference test (T-00) was executed without EPS to investigate the positive projection installation mechanism and its effect on the surrounding pressure above and adjacent to the culvert, as shown in Fig. 6. The results indicate that the vertical pressure measured at the culvert centerline (MPS1) significantly increased with the applied load. However, the pressure far from the edge of the culvert by a distance equals to Bc (MPS2) remained approximately the same as the applied pressure. At an applied pressure of 20 kPa, the recorded pressures were approximately 34 kPa at MPS1 and 18.5 kPa at MPS2. As the applied load increased to 80 kPa, the pressure above the culvert reached approximately 135 kPa, whereas the pressure in the surrounding soil was approximately 82 kPa. At the maximum applied pressure of 140 kPa, the recorded pressure at MPS1 increased to nearly 230 kPa, while the pressure at MPS2 was approximately 155 kPa. These results demonstrate a significant stress concentration above the rigid culvert, where the pressure measured at MPS1 was approximately 40%–50% higher than that measured in the surrounding soil under high loading levels. This behavior indicates the mobilization of negative soil arching, which occurs because the rigid culvert undergoes smaller settlement compared with the surrounding soil mass. Consequently, a greater portion of the applied load is transferred to the soil column directly above the culvert, resulting in increased vertical stresses on the culvert top.

### Effect of EPS inclusion with different densities

**Fig. 7:**
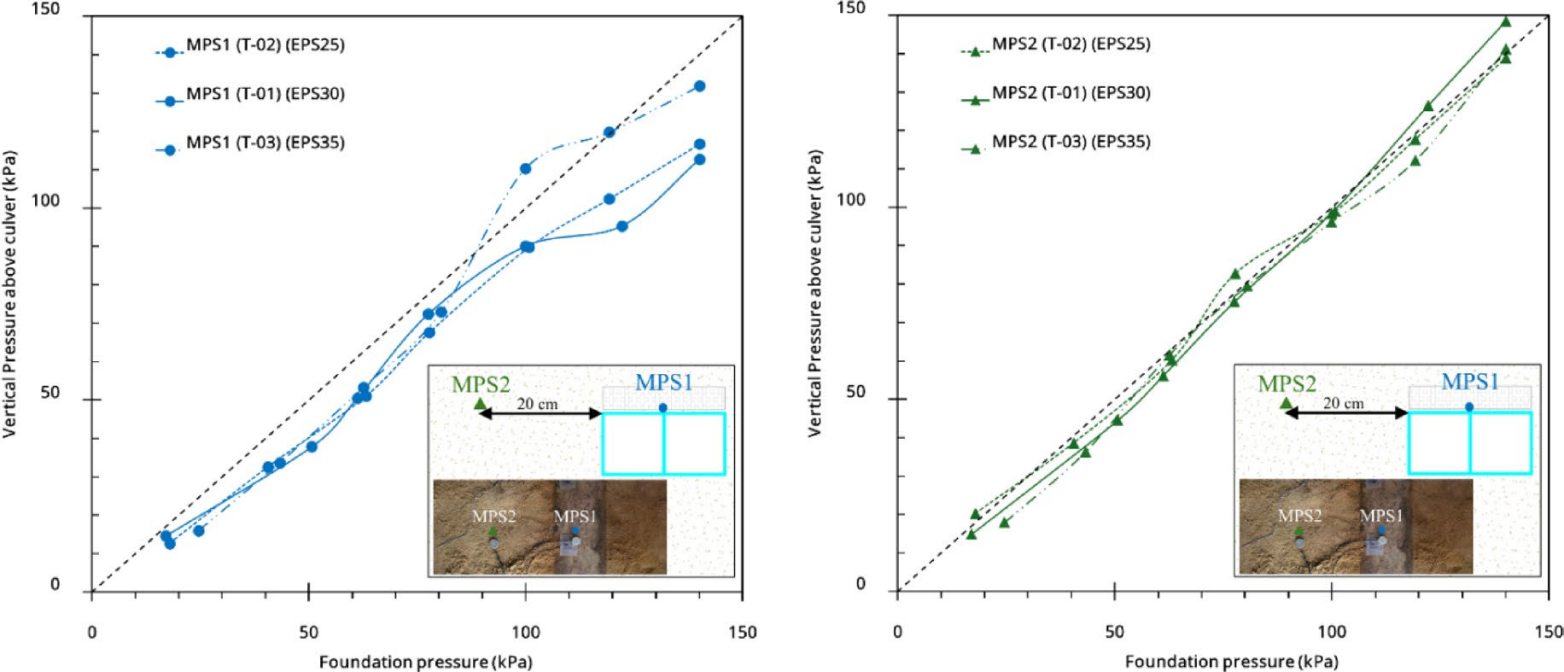
Pressure distribution at and beside the culvert for tests T-01, T-02, and T-03.

To investigate the implementation of the induced trench installation (ITI) method, the vertical pressure distribution above the culvert with EPS geofoam inclusion with three different densities was examined, as shown in Fig. 7. The results correspond to tests T-01, T-02, and T-03, which utilized geofoam densities of 30, 25, and 35 kg/m³, respectively. In all tests, the geofoam thickness and width were equal to 0.125 Bc (2.5 cm) and Bc (20 cm), respectively. The EPS inclusions were directly positioned above the culvert top. The results indicate that the three EPS densities exhibited similar trends with increasing applied foundation pressure. By increasing the applied pressure, the measured vertical pressure above the culvert increased. Despite this, the measured pressure above the culvert remained lower than the applied pressure. At an applied pressure of 140 kPa, the measured vertical pressures above the culvert were approximately 113 kPa, 116 kPa, and 131 kPa for EPS densities of 25, 30, and 35 kg/m³, respectively. This indicates that EPS inclusion reduced the vertical pressure acting on the culvert by approximately 15% on average compared with the applied load. The pressure measured at MPS2 is illustrated in Fig. 7. The results show that the measured pressures at this location were approximately equal to the applied foundation pressure. This observation indicates that the influence of the differential movement between the soil above the culvert and the surrounding soil became negligible beyond a horizontal distance approximately equal to the culvert width, which is in line with the findings of^10^. Although the three densities exhibited comparable behavior, slightly higher pressures were observed for the higher-density geofoam (EPS-35), particularly at higher loading levels. This behavior is due to the increased stiffness of higher-density EPS, which limits compressibility and reduces the effectiveness of stress redistribution. On the other hand, lower-density EPS is more compressible, which causes the soil prism above the culvert to settle differently than the soil columns around it. This makes positive soil arching more likely to occur. The results confirm that EPS geofoam inclusion initiates the mobilization of positive soil arching, resulting in a redistribution of stresses from the culvert top toward the surrounding soil mass. The magnitude of this effect is influenced by the stiffness contrast between the geofoam and backfill soil.

### Effect of EPS thickness

**Fig. 8:**
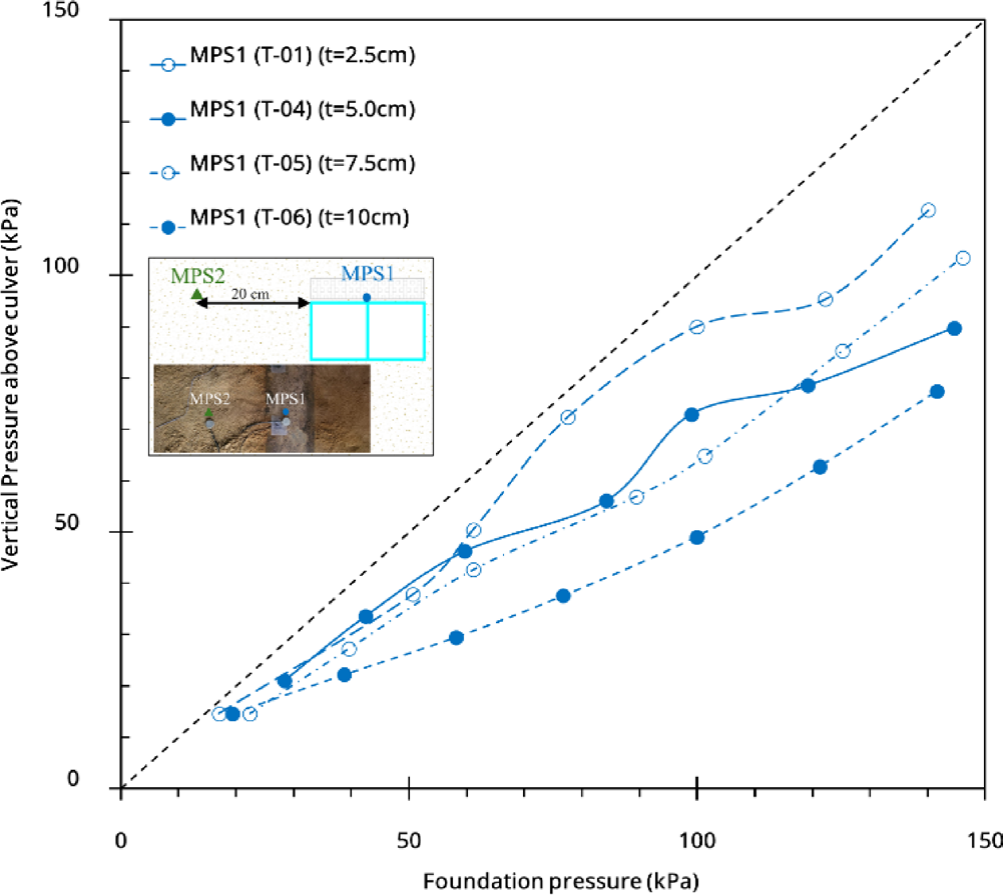
Pressure distribution above the culvert for tests T-01, T-04, T-05, and T-06.

The influence of the EPS inclusion thickness was investigated by considering four different thicknesses. The EPS inclusion width was constant for all tests and equal to the culvert width Bc. In addition, the density of the EPS inclusion was 30 kg/m³ in all tests. Furthermore, the EPS inclusions were placed directly above the culvert. Figure 8 compares the vertical pressure measured above the culvert centerline (MPS1) for tests T-01, T-04, T-05, and T-06, where the EPS geofoam thicknesses were 2.5 cm (0.125Bc), 5.0 cm (0.25Bc), 7.5 cm (≈ 0.375Bc), and 10.0 cm (0.5Bc), respectively. The results indicated that the vertical pressure above the culvert increased as the applied foundation pressure increased for all tests. At relatively low applied pressures, from 20 to 50 kPa, the responses of tests T-01, T-04, and T-05 were generally similar. Whereas for test (T-06) exhibited a noticeably lower pressure response, starting at approximately 30 kPa, indicating a more effective stress redistribution. The measured reductions in pressure for tests T-01, T-04, T-05, and T-06 compared with the applied foundation pressure were approximately 16%, 29%, 33%, and 45%, respectively, which confirms that increasing the thickness of the EPS inclusion significantly enhanced the reduction in the vertical stress acting on the culvert. This is due to the fact that thicker compressible layer allows for greater differential settlement between the soil column above the culvert and adjacent to the culvert. Which promotes the development of positive soil arching and transfers a larger portion of the applied load to the adjacent soil columns.

### Effect of EPS inclusion location

**Fig. 9:**
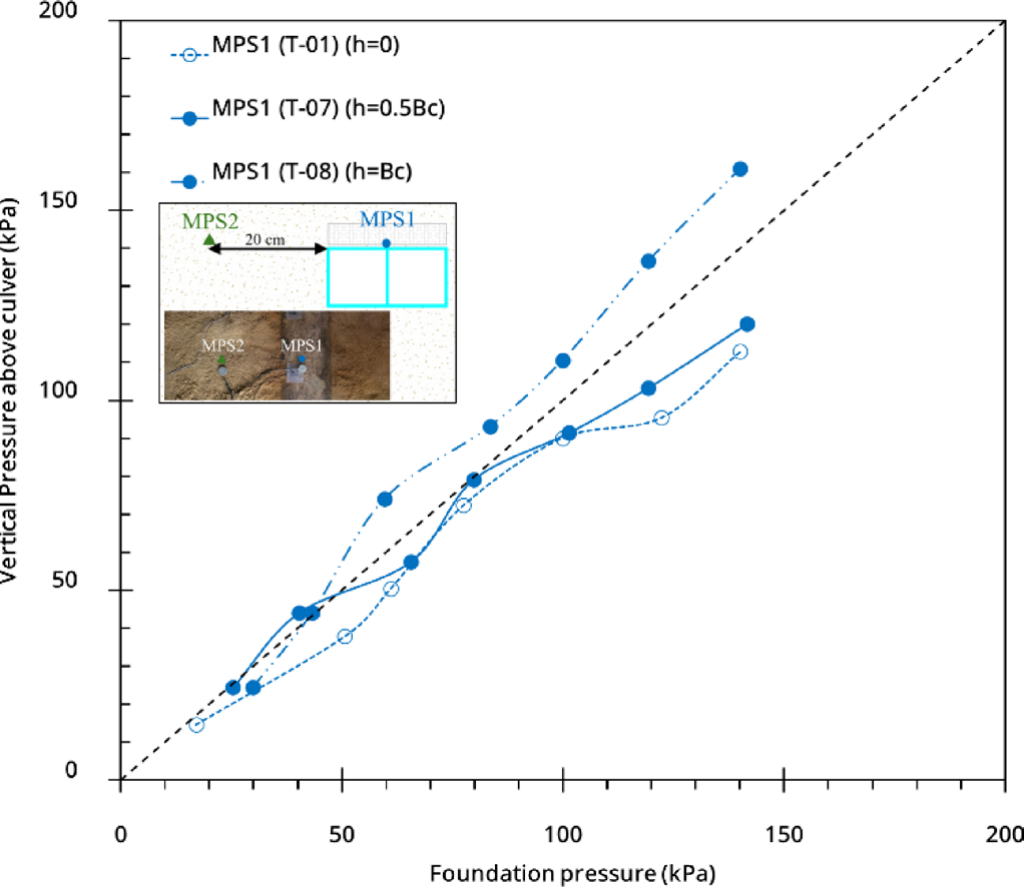
Pressure distribution above the culvert for tests T-01, T-07, and T-08.

Figure 9 shows the vertical pressure measured at the culvert centerline (MPS1) for tests T-01, T-07, and T-08, where EPS geofoam with the same density (EPS30) and width equal to the culvert width (Bc = 20 cm) was used, while the vertical distance between the EPS inclusion and the culvert top was varied. The EPS layer was installed directly above the culvert (0.0 cm) in test T-01, at 10 cm (0.5Bc) in test T-07, and at 20 cm (Bc) in test T-08. The results show that the vertical pressure above the culvert increased with the applied foundation pressure for all tests. Furthermore, the position of the EPS inclusion significantly influenced the magnitude of the pressure transmitted to the culvert. When the EPS inclusion was installed directly above the culvert the measured vertical pressures were considerably lower than those recorded in the tests where the EPS layer was positioned far from the culvert top. Compared with the applied foundation pressure, the reductions were approximately 16% for T-01 and 7% for T-07, whereas for T-08, the measured pressure above the culvert slightly exceeded the applied pressure at higher loading levels. This behavior indicates that increasing the distance between the EPS inclusion and culvert reduces the effectiveness of stress redistribution. Because the EPS layer was installed directly above the culvert, the compressible inclusion interacted directly with the soil column responsible for the load transfer, which promoted the mobilization of positive soil arching and reduced the vertical stress acting on the culvert. However, when the EPS inclusion is located far above the culvert, the differential movement between the soil columns becomes insufficient to effectively mobilize the soil arching resulting in higher pressures being transmitted to the structure. These findings demonstrate that the most effective location for the EPS inclusion is directly above the culvert, where the compressible layer can maximize stress redistribution within the embankment. The results obtained in this study are consistent with those of^10^.

### Effect of EPS width

**Fig. 10:**
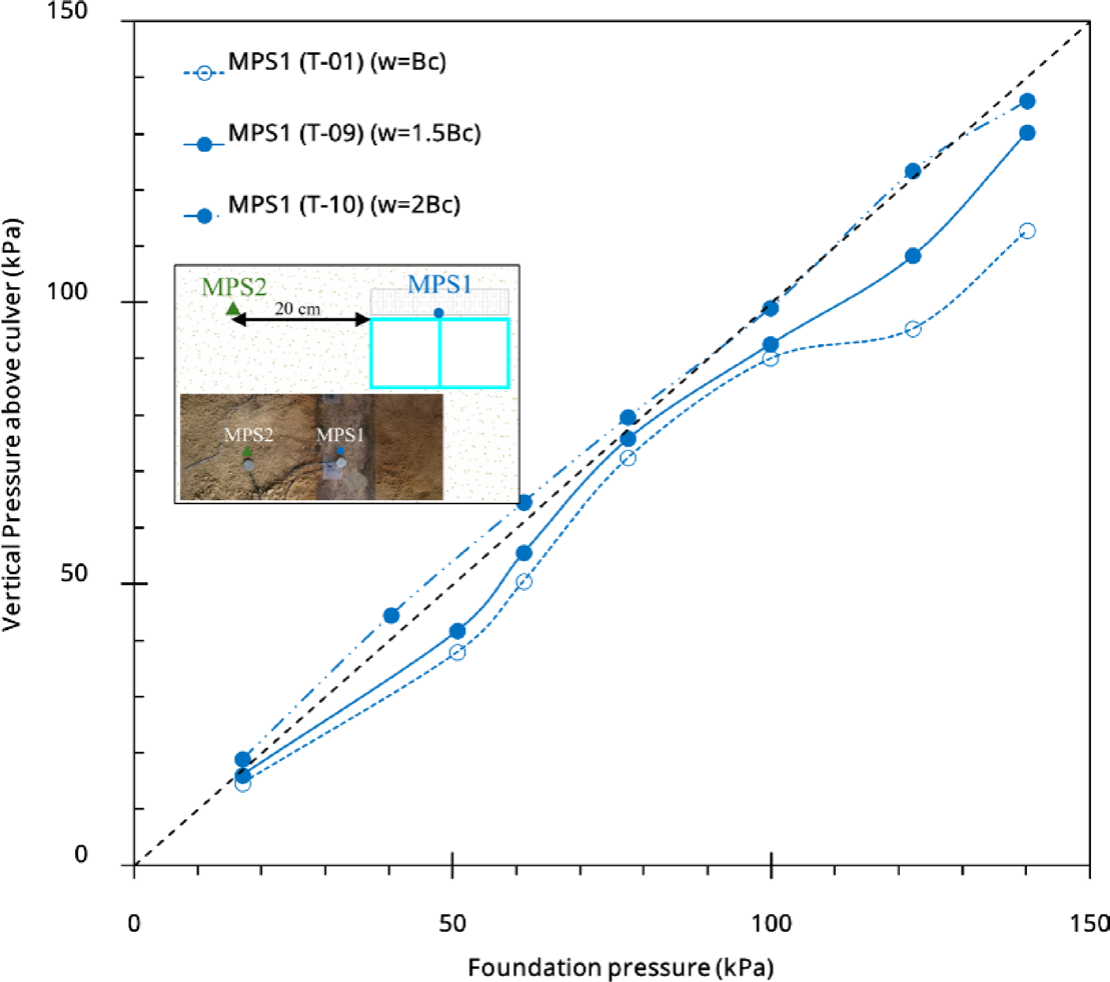
Pressure distribution above the culvert for tests T-01, T-09, and T-10.

Figure 10 shows the vertical pressure measured at the culvert centerline (MPS1) for tests T-01, T-09, and T-10, where EPS geofoam with the same density (EPS30) and thickness (0.125Bc = 2.5 cm) was used, while the inclusion width was varied. The investigated widths were Bc (20 cm), 1.5Bc (30 cm), and 2Bc (40 cm) for tests T-01, T-09, and T-10, respectively. The results indicate that the vertical pressure above the culvert increased with increasing applied foundation pressure for all tests. Among the investigated configurations, T-01, where the EPS width was equal to the culvert width (Bc), produced the greatest reduction in the vertical pressure above the culvert, approximately 16% of the applied pressure. While for tests T-09 and T-10, the amount of pressure that reached the top of the culvert was approximately equal to the applied pressure. This behavior can be explained by the fact that when the width of the EPS inclusion is equal to the culvert width, a clear differential settlement develops between the soil column above the culvert and the adjacent soil columns, which promotes the development of soil arching. However, as the EPS width increased beyond the culvert width, the compressible zone extended into the surrounding soil region reducing the differential settlement required to mobilize soil arching and consequently decreasing the stress reduction efficiency.

## Discussion

**Fig. 11:**
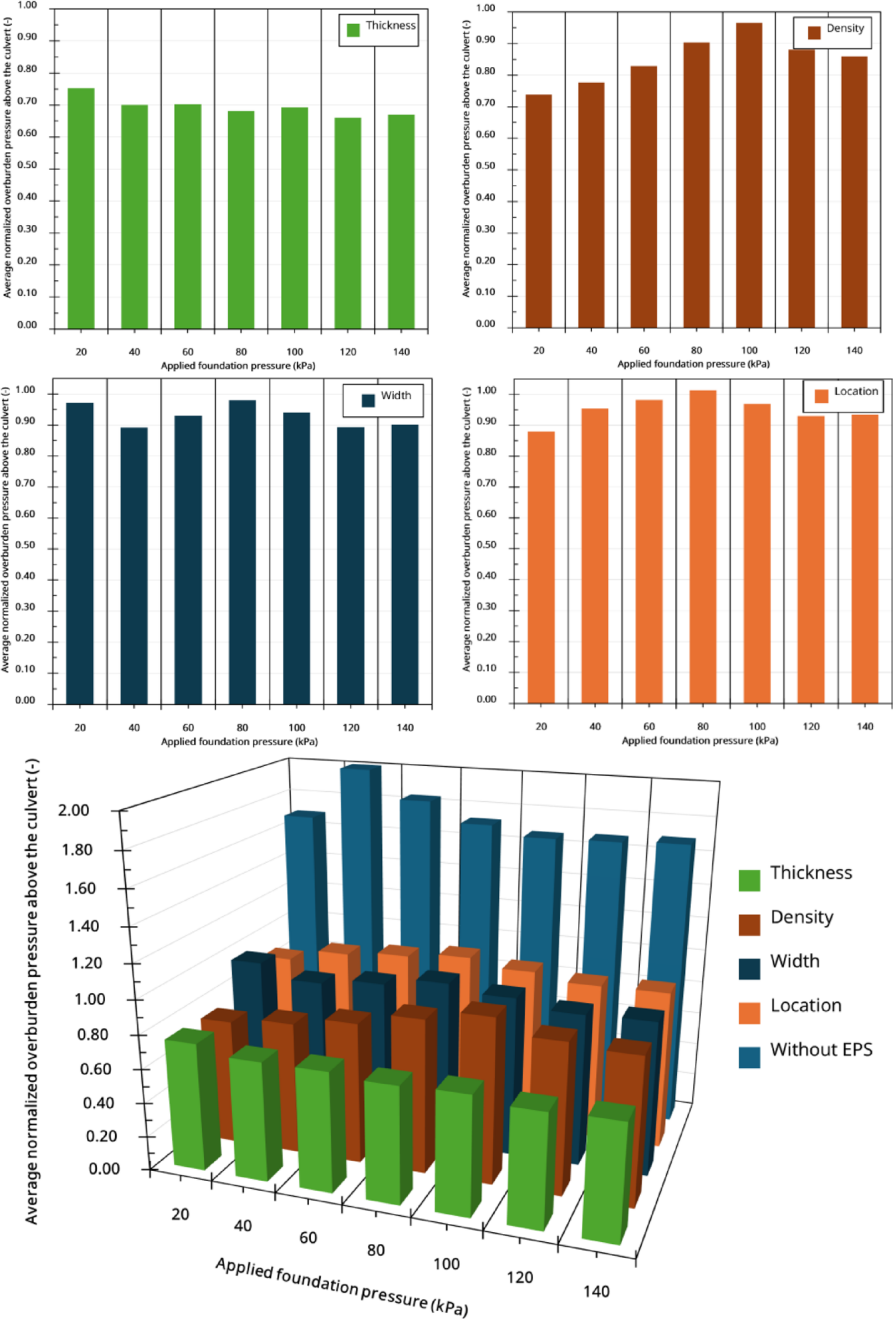
Variation of normalized vertical pressure above the culvert with applied foundation pressure for different EPS inclusion parameters.

The experimental results indicated that the installation of the EPS geofoam significantly changes the load transfer mechanism within the soil mass above the buried rigid culvert compared with the reference condition T-00 without the presence of the EPS. In test T-00, the measured pressure above the culvert exceeded the applied foundation pressure. This phenomenon occurs because negative soil arching develops as a result of the higher stiffness of the rigid culvert compared to the surrounding backfill. Thus, the stiffness contrast controlled the settlement of the soil column directly above the culvert, causing a concentration of vertical stresses on the culvert top. However, installing the EPS geofoam created a compressible inclusion within the soil prism above the structure. This modification changed the stiffness distribution and induced differential settlement between the central soil column and the adjacent soil masses. Consequently, the observed stress redistribution within the backfill and the reduction in vertical pressure above the culvert are indicative of the development of positive soil arching. It should be noted that this interpretation is based on the measured pressure distribution within the backfill, as direct measurements of settlement, EPS compression, or soil deformation were not included in the experimental program. The comparative evaluation presented in Fig. 11 shows that the normalized pressure acting on the culvert decreased noticeably for all investigated EPS configurations compared with the test without EPS. Since the normalized pressure represents the ratio between the measured pressure directly above the culvert at MPS1 and the applied foundation pressure. Figure 11 indicated that the thickness of the EPS inclusion had the most influence on the stress reduction among the investigated parameters. Increasing the EPS thickness significantly increases the compressibility of the inclusion layer and allows greater relative deformation of the soil column above the culver which is facilitating the mobilization of soil arching. As a result, the average normalized pressure above the culvert was reduced to approximately 0.70 of the applied pressure. In contrast, the effect of EPS density has the second influence on the stress reduction within the investigated parameters. Lower EPS density produced slightly lower pressures above the culvert due to its higher compressibility, whereas higher EPS density behaved more rigidly, limiting deformation and consequently reducing the effectiveness of stress redistribution. Changing in the EPS density indicated average normalized pressure above the culvert in the range of 0.8 of the applied pressure.

The influence of the EPS inclusion width was also evident in the results. Within the investigated parameters, the effective configuration for EPS was noticed when its width was equal to the culvert width. In this configuration, the compressible zone was equal to the soil column directly above the culvert which maximized the differential settlement required to mobilize soil arching. However, when the EPS width extended beyond the culvert width, the stiffness contrast between the central and adjacent soil columns was reduced and consequently diminished the efficiency of stress reduction. Furthermore, the location of the EPS inclusion relative to the culvert top significantly influenced the overburden pressure reduction above the culvert. The largest stress reduction occurred when the EPS layer was placed directly above the culvert. Since increasing the distance between the EPS inclusion and the culvert reduced the interaction between the compressible layer and the soil column responsible for load transfer. Consequently, the soil arching mechanism became weaker and the vertical pressure acting on the culvert increased.

**Fig. 12:**
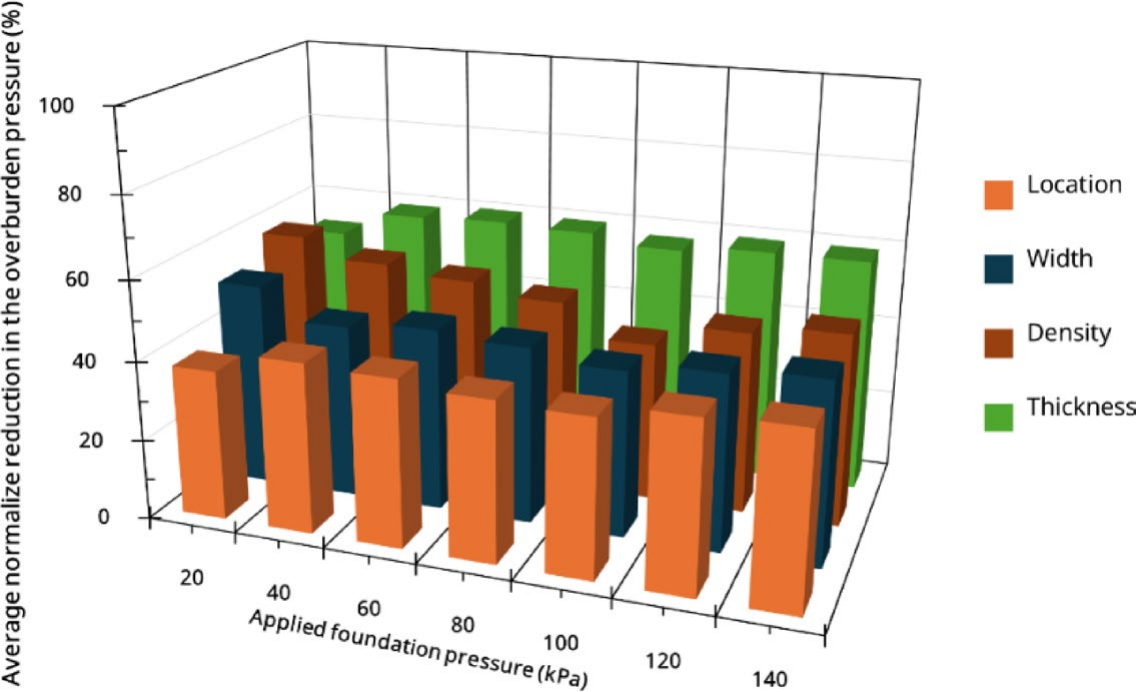
Comparison of pressure reduction efficiency for different EPS inclusion parameters relative to the reference condition (T-00).

Figure 12 shows the average normalized reduction in the overburden pressure measured directly above the culvert at MPS1 compared with that measured in the reference test. The pressure reduction relative to the reference test reached approximately 50% and 60% for the EPS thickness, the reductions associated with EPS density ranged between approximately 40% and 60%, the reductions associated with EPS width ranged between approximately 40% and 50%, and the EPS location parameter exhibited the smallest reduction values ranging between 40% and 45%.

The results indicate that the effectiveness of EPS geofoam in reducing the vertical pressure acting on buried rigid culverts is primarily governed by its ability to induce differential settlement within the soil mass between the soil column above and adjacent to the culvert top and thereby induce the soil arching. Among the investigated parameters, EPS thickness plays the dominant role in controlling the magnitude of stress reduction, then the EPS density. Contrarily, the EPS width and location exhibit lower effects compared to the former EPS parameters in governing the degree of interaction between the compressible layer and the soil column above and adjacent to the culvert. The previous findings highlight the importance of optimizing the geometric configuration, density, and location of EPS inclusions in induced trench installation to maximize the soil arching and improve the structural performance under high embankment backfill above buried rigid culverts.

## Conclusion

This study experimentally investigated the influence of EPS geofoam inclusion parameters on the vertical stress distribution above buried rigid box culverts installed using the induced trench method. Based on the conducted laboratory tests, the following could be concluded:

1. The reference test performed without EPS geofoam demonstrated a pronounced concentration of vertical stress above the culvert. The measured pressure at the culvert centerline exceeded the applied foundation pressure by approximately 60% to 70% at high loading levels, confirming the development of negative soil arching caused by the higher stiffness of the rigid culvert relative to the surrounding soil.
2. Placing EPS geofoam above the culvert significantly affected the load transfer mechanism within the backfill. Since the EPS inclusion modified the stiffness distribution within the soil mass and induced differential settlement between the soil column above and adjacent to the top of the culvert. This mechanism confirmed the mobilization of positive soil arching, which redistributed a portion of the applied load away from the culvert.
3. EPS thickness was found to be the most influential factor in controlling stress reduction within the investigated parameters. Increasing the EPS thickness from 2.5 cm to 10 cm increased the pressure reduction above the culvert from approximately 50% to 70% relative to the reference test (T-00).
4. The density of EPS geofoam indicated the second influence factor after the EPS thickness on stress redistribution. Although the change in the EPS density slightly improved the pressure reduction, the lower EPS density exhibited a slightly greater pressure reduction due to its higher compressibility, whereas a higher EPS density behaved more rigidly and consequently reduced the efficiency of stress mitigation.
5. The geometric configuration and placement of the EPS inclusion also affected the effectiveness of stress redistribution. Within the investigated experimental range, the greatest stress reduction was observed when the EPS width was equal to the culvert width and the geofoam layer was placed directly above the culvert. Increasing either the EPS width beyond the culvert width or the vertical distance between the EPS layer and culvert reduced the differential settlement and hence the mobilization of the soil arching.

The experimental findings highlight the importance of selecting the thickness, density, width and placement configuration of EPS geofoam when used as a compressible inclusion in induced trench installations. Overall the EPS geofoam can effectively reduce the vertical stresses acting on buried rigid culverts and improve their structural performance under high embankment loading conditions.

## Data Availability

The data presented in this study are available in the article.

## Abbreviations

EPS: Expanded polystyrene
ITI: Induced trench installation
Bc: Culvert width
Hc: Culvert height
ASTM: American Society for Testing and Materials
MPS: Miniature pressure cell
